# Infantile Paralysis

**Published:** 1856-06

**Authors:** Wm. Adams


### ON INFANTILE PARALYSIS.

BY WM. ADAMS.

Whether paralysis of particular muscles or limbs, independent of traumatic lesion, is ever congenital, Mr. Adams considers to be at least doubtful. The cases related of limbs remaining flaccid and useless in infants born asphyxiated, after difficult and instrumental labors, and of facial paralysis usually of one side, and sometimes accompanied with loss of power in the corresponding arm, etc., which had in some instances been satisfactorily traced to traumatic lesion—cannot be admitted as examples of the affection described. Infantile paralysis usually occurs between the ages of six and eighteen months, generally during difficult dentition, and often preceded by fits or convulsions. It may, however, occur at earlier, or at later periods. In one of Mr. Adams' cases, it occurred at the age of five years; and both arms, as well as both legs, were paralyzed. It is said frequently to happen without any convulsive disorder, and when the children are in robust health. Mr. Adams, however, considers that in many of these cases the children had fits, which passed away unnoticed in the night; and careful inquiry had convinced him that in most cases the children were at the time suffering from a slight febrile condition. Many children, apparently in good health, became heated and feverish during the night; the skin, especially of the face, being hot and burning, and the head freely perspiring. Paralysis in children may result from intestinal irritation caused by worms, indigestible food, etc. The cause may be either centric or eccentric irritation. It not unfrequently follows marked febrile disorders, especially measles and whoopingcough. It is the author's opinion that where many muscles or entire limbs are affected, and where paralysis is persistent, it depends upon structural lesion of the nervous centers, brain or spinal cord; that in similar cases, in which the paralysis is transient, it depends upon congestion of the nervous centers, sometimes accompanied with effusion, which afterwards becomes absorbed; and that where single muscles, or a group of associated muscles, are affected, it depends upon some local failure of nutrition in the nerves supplying the muscles, under a general, though perhaps slight, febrile condition. M. Bouchut describes this affection under the title of "myogenic or essential paralysis" ("Practical Treatise on the Diseases of Children," translated by Mr. P. H. Bird;) and admits, as a cause, lesion of the nervous centers and cords only in those cases which succeed febrile convulsions. The other cases he groups in two classes; viz: those accompanied with pain in the affected limb, and those following convulsions without febrile excitement; and in these he considers the cause to be primarily and essentially an alteration of the elementary tissue of the substance of the muscles. The nature of the affection in these cases he regards as "entirely rheumatic," and traces it as a frequent result of exposure to cold. Mr. Adams has not seen any cases accompanied with pain; but, upon the ground of deficient evidence, he doubts the rheumatic character of the affection under any circumstances, and regards it as probable that the children who, in restless nights, throw off the bedclothes, are frequently suffering from febrile or eccentric irritation. No evidence is given of alteration in the elementary structure of the muscles in the early stages; and Mr. Adams considers the myogenic theory to be advanced without sufficient evidence. M. Bouchut states that the development of the paralysis is usually slow. It is the author's experience, it had always been sudden; and it is considered that, in the cases of supposed slow development, the consecutive phenomena—contraction and atrophy—had taken place. In these cases, the limb is often said to get weaker; when it occurs in the leg, the lameness increases, but this is due to the supervention of contraction, and not to any increase of the paralytic affection, which, indeed, is not unfrequently improving. M. Bouchut observes that, "whether at the beginning or at the end of the myogenic paralysis, sensation remains quite perfect." In this the author entirely concurs. Mr. Adams has also noticed that there does not appear to be any disposition, in the paralyzed muscles to become rigid, as in cases of adult paralysis recently noticed more particularly by Dr. Todd. The muscles either remain flaccid throughout life, or, by the spontaneous disappearance of the paralysis, they are restored to a healthy condition; or complete recovery is arrested, and the muscles remain partially paralyzed through life. This latter is believed to be the most frequent termination; the complete recovery second; and the persistent flaccid condition third, in relative frequency. The paralysis most commonly affects some of the muscles of one leg; very frequently the leg and arm of the same side; occasionally both legs; and very rarely both legs and both arms. When single muscles are affected, the most frequent to suffer are—1, the extensor longus digitorum of the toes; 2, the tibialis anticus; 3, the deltoid; 4, the sterno mastoid. When particular groups of muscles are affected, the most frequent to suffer are—1, those on the anterior part of the leg, forming the extensors of the toes and flexors of the foot; 2, the extensors and supinators of the hand, always together; 3, the extensors of the leg, and with them generally the muscles of the foot in the first class. At the time of seizure, the author is unable to say whether any other muscles were affected; but if so, they completely recovered, as in the last stage the cases presented well-marked examples of paralysis of single muscles or groups of muscles. Sir B. Brodie lately mentioned to the author a case brought to him in which the muscles of deglutition were paralyzed in a child. The attempts to swallow were very painful to witness. He did not know the result, but death from starvation probably took place. In the Royal Orthopœdic Hospital, where these cases apply in considerable numbers, no case had been seen in which the muscles of the hip-joint were involved. Some patients, in whom both legs were affected, the rectus and other muscles of the thighs, as well as those of the legs being paralyzed, have never walked at all; but the existence of power in the muscles of the hip-joints, enables us to make these patients walk, by mechanically fixing the knee and ankle-joints, with considerable freedom. This affection exhibits a strong tendency towards spontaneous cure. In some cases, the paralysis completely disappears, even when entire limbs are involved; but in reference to severe cases, Mr. Adams believes with Sir B. Brodie, that unless recovery takes place within a few months, the paralysis is generally persistent through life. In slight and moderately severe cases, the rule is, that either complete recovery or very great improvement takes place; and this frequently several years after the seizure. Numerous cases are seen at the Orthopœdic Hospital in all stages of spontaneous recovery. The second stage is marked by deformity, produced by adapted atrophy of certain muscles, determined by paralysis of the opponent muscles and position of the part, as seen in the commonest form—elevation of the heel. The author advises the removal of the contraction in the lower extremeties by division of the tendons, whenever it interferes with the motions of the joints necessary to progression and the erect position. Loss of power can be subsequently compensated for to a great extent by mechanical means, the joints being either rendered available in progression, or fixed. Infantile paralysis lays the foundation of a very large proportion of all the non-congenital deformities, itself being frequently only a transient condition. If the mode of production of these deformities were rightly understood, their prevention would be easy. Passive muscular exercises, according to the circumstances of the case, and properly adapted mechanical supports, are the preventive measures indicated. In the medical treatment, gentle mercurials for a few months after the seizure are recommended, if not injurious to the general health, but, beyond this period, any internal remedies, except those calculated to improve the general health, are of little use. Febrile irritation must be allayed; and in difficult dentition the gums may be lanced. Although this cannot remove the mischief, it may contribute to this end, and diminish its effects. Mr. Adams has not seen benefit from blisters or other counter-irritants, though he had used them. He prefers shampooing, galvanism, warm clothing, sea-bathing, and passive exercises, as likely to aid the vigorous and frequently successful efforts made by nature. The hæmospastic apparatus invented by Dr. Junod was very useful in maintaining a natural temperature in the paralytic extremities. To some extent the apparatus had been useful in keeping a good supply of blood in the muscles, and preventing atrophy.—*Ranking's Abstract.*

